# Immersion-Based *Candida albicans* Infection in Adult Zebrafish Induces Distinct Behavioral, Immune and Oxidative Responses in a Dose- and Time-Dependent Manner

**DOI:** 10.1007/s00284-026-05109-4

**Published:** 2026-08-26

**Authors:** Marcos Otávio Bueno, Ricardo Cervini, Vinicius Cataldo Ferraz, Adriano Tony Ramos, Claudriana Locatelli, Ariana Centa, Gustavo Colombo Dal-Pont, João Paulo Assolini

**Affiliations:** 1 Universidade Alto Vale do Rio do Peixe (UNIARP), Rua Victor Baptista Adami, 800, Caçador, Santa Catarina 89500-000 Brazil; 2 Experimental Pathophysiology Research Group, Universidade Alto Vale do Rio do Peixe (UNIARP), Caçador, Santa Catarina Brazil; 3 Translational Health Research Group, Universidade Alto Vale do Rio do Peixe (UNIARP), Caçador, Santa Catarina Brazil; 4 Graduate Program in Development and Society, Universidade Alto Vale do Rio do Peixe, Caçador, Brazil; 5 Federal University of Santa Catarina (UFSC), Curitibanos, Santa Catarina Brazil

## Abstract

Candidiasis caused by *Candida albicans* represents a major opportunistic infection, particularly in hospitalized individuals, with high morbidity and mortality rates. Experimental models that reproduce natural routes of exposure and allow integrated analyses of host responses remain limited. In this study, we established and characterized an immersion-based infection model of *C. albicans* in adult zebrafish (*Danio rerio*) to investigate systemic, behavioral, immunological, oxidative, and histopathological outcomes. Adult zebrafish were exposed by immersion to different fungal concentrations (1 × 10⁶ or 1 × 10⁸ CFU/mL) for 10–24 h. Fungal burden, exploratory behavior, antioxidant capacity (ABTS), TNF-α levels, and tissue alterations were subsequently assessed. Exposure to 1 × 10⁸ CFU/mL for 24 h resulted in a significant increase in fungal burden without inducing mortality, characterizing a sublethal infection. Infection modulated behavioral parameters, tissue-specific alterations in antioxidant activity and a marked reduction of TNF-α levels in the liver and brain were observed, suggesting disruption of immune-redox homeostasis. Histopathological analyses revealed mild inflammatory responses and increased melanomacrophage accumulation, particularly in skin and gills tissues. Overall, this study demonstrates that adult zebrafish immersion constitutes a biologically relevant and translational model for investigating candidiasis, enabling integrated evaluation of host–pathogen interactions and systemic responses under environmentally relevant conditions.

## Introduction

Candidiasis caused by *Candida albicans* is one of the leading opportunistic infections among hospitalized individuals, frequently associated with clinical conditions that compromise physical barriers, such as invasive surgeries, extensive burns, and prolonged catheter use, as well as with biofilm formation on medical surfaces. As a result, it represents a major cause of bloodstream infections, with mortality rates exceeding 40% [1, 2]. *C. albicans* and non-*albicans* species are dimorphic fungi capable of switching between yeast and hyphal forms, a key virulence mechanism that enables adaptation to different niches according to temperature and macronutrient availability, thereby facilitating the transition from a commensal to a pathogenic state [3, 4]. In addition to morphological switching, the pathogenesis of candidiasis involves multiple virulence factors, including increased expression of adhesion molecules, biofilm formation on host cells, secretion of hydrolytic enzymes, phenotypic and genotypic alterations, and immune evasion mechanisms [5, 6]. Clinically, infections caused by *Candida* species may disseminate and affect deep organs, compromising systems such as the urinary and digestive tracts as well as the central nervous system [7, 8].

Given the complexity of the virulence mechanisms of *Candida* spp. and the clinical impact of these infections, the use of experimental models that allow integrated investigation of immunological, pathophysiological, and behavioral aspects of infection is essential [9]. In this context, the zebrafish (*Danio rerio*) stands out as a versatile and highly translational tool. Approximately 70% of human genes have protein-coding orthologs in the zebrafish genome, enabling extrapolation of findings from studies addressing human physiology and pathology [10]. In addition, the ease of animal maintenance and handling, requiring limited space and low feeding costs, represents a major advantage over other animal models, characterizing zebrafish as a safe, efficient, and cost-effective model for contemporary biomedical research [11].

Experimental models used to study *Candida albicans* infections are mainly focused on mammals or on zebrafish larvae subjected to microinjection, approaches that only partially reproduce natural exposure mechanisms and may limit the assessment of behavioral and systemic outcomes [12]. Although widely employed, microinjection induces infection through an artificial and invasive route, failing to reflect physiological colonization pathways such as the skin, mucosal surfaces, or gills [13]. Moreover, zebrafish larvae do not possess a fully developed immune system, which restricts integrated investigations of innate and adaptive immunity [14].

Zebrafish infection models have demonstrated that different exposure routes can distinctly modulate the innate immune response. Immersion-based infection of germ-free larvae with *Listeria monocytogenes*, although non-lethal, was sufficient to induce the expression of inflammatory genes [15]. Similarly, both immersion and microinjection have been employed to infect zebrafish larvae and adults, with immersion representing a simple and efficient procedure for mutant or drug screening, capable of activating inflammatory markers in individual embryos [16, 17, 18].

Furthermore, comparative studies have shown that different infection routes can trigger distinct sets of innate immune genes, as observed for *Vibrio parahaemolyticus*, suggesting that each method elicits specific and complementary immune responses [19]. This principle also applies to *Borrelia burgdorferi* infection, for which immersion- and microinjection-based protocols in zebrafish larvae have been established as practical, high-throughput in vivo models with potential to investigate pathogenic mechanisms and to test antibiotic therapies against resistant forms of the pathogen [20].

Although studies in larvae highlight the impact of different infection routes on innate immunity, this model does not allow a comprehensive evaluation of the systemic processes observed in organisms with a fully developed immune system, underscoring the need to investigate these interactions in adult zebrafish. Immersion-based infection represents an ecologically relevant method capable of mimicking real exposure conditions and allowing the pathogen to initially interact with epithelial barriers, thereby more closely reproducing natural pathophysiological processes. Accordingly, adult-based immersion models may provide a more suitable approach for exploring the systemic effects of fungal infection, including behavioral, immunological, and biochemical alterations.

Thus, the aim of this study was to evaluate the effects of *Candida albicans* infection in adult zebrafish exposed by immersion, by analyzing fungal burden, behavioral alterations, immunological responses, oxidative parameters, and histopathological changes across different exposure concentrations and time points.

## Methods

### Animals

Adult zebrafish (*Danio rerio*) were obtained from specialized commercial suppliers in the midwestern region of Santa Catarina, Brazil. A total of 100 specimens, approximately 3 cm in length, were transported to the Higher Education Research Laboratory at the Universidade Alto Vale do Rio do Peixe (UNIARP), where they underwent an acclimation period prior to experimentation. The animals were distributed into five 4-L aquaria, with 20 individuals per tank, following methodological recommendations described by Bernardo et al. [21] and Baggio et al. [22].

Fish were acclimated for eight days under controlled temperature (25 ± 2 °C) and a 14 h light/10 h dark photoperiod. The aquaria were maintained under continuous recirculation and aeration, and the fish were fed once daily with commercial fry feed. Water physicochemical parameters, including pH (7.0 ± 1) and chlorine levels, were regularly monitored throughout the acclimation period. Animals were observed daily, and only healthy fish without visible lesions or abnormal behavior were included in the study.

### Maintenance and Preparation of the *Candida albicans* Inoculum

*Candida albicans* strain NEWP 0031 (Newprov, Brazil; lot no. 64037; code PA249) was used in this study. The *C. albicans* strain was initially activated in Brain Heart Infusion (BHI) broth and incubated at 36 °C to allow metabolic recovery. Subsequently, the cells were cultured on Sabouraud dextrose agar, also at 36 °C, ensuring adequate growth and preservation of the species’ morphological characteristics. For preparation of the experimental inoculum, fresh colonies were collected from Sabouraud agar plates and resuspended in PBS. Cell concentration was adjusted based on optical density (OD), according to the methodology described by Wang et al. [23], allowing the preparation of homogeneous suspensions at the desired concentrations for infection assays.

### Experimental Infection of Zebrafish with *Candida albicans*

Zebrafish infection was conducted according to a protocol adapted from Kuo et al. [24]. *Candida albicans* suspensions at concentrations of 1 × 10⁶ CFU/mL or 1 × 10⁸ CFU/mL were added directly to aquaria containing 4 L of water, allowing immersion exposure of the animals for either 10–24 h. Accordingly, five experimental groups were established: control (uninfected), 1 × 10⁶ CFU/mL for 10 min, 1 × 10⁶ CFU/mL for 24 h, 1 × 10⁸ CFU/mL for 10 min, and 1 × 10⁸ CFU/mL for 24 h. After completion of each exposure period, aquarium water was completely renewed to remove residual fungal inoculum. Subsequently, all animals were maintained under standard husbandry conditions for an additional 8 days. At the end of the post-exposure period, zebrafish were first subjected to behavioral analyses. Following behavioral assessment, animals were euthanized and tissues were collected for biochemical and microbiological analyses.

### Sample Collection and Euthanasia

After completion of the experiments, the fish were euthanized by rapid cooling through immersion in ice-cold water (0–4 °C), in accordance with ethical recommendations for small fish species. The animals remained immersed for approximately 10 min until complete cessation of opercular movements, which was used as the criterion to confirm death. Subsequently, dissections were performed for collection of the brain and liver, which were immediately processed or stored according to the requirements of the subsequent assays.

### Preparation of Tissue Homogenates

Brain and liver samples collected from zebrafish in each experimental group were combined to generate three independent biological pools per group, due to the limited amount of tissue obtained from individual animals. Each pool was homogenized in 20 mM sodium phosphate buffer (pH 7.4) containing 0.1% Triton X-100 and 150 mM sodium chloride, at a 1:10 (w/v) ratio, supplemented with protease inhibitor. Homogenization was performed on ice (4 °C) using approximately 20 strokes with a manual homogenizer, followed by centrifugation at 10,000 rpm for 10 min, also at 4 °C. The resulting supernatants were either used immediately for analyses or stored at − 80 °C until use. Protein concentration in the supernatants was determined spectrophotometrically using a NanoDrop system. The prepared homogenates were subsequently used for the determination of antioxidant activity by the ABTS method and for quantification of TNF-α levels.

### Colony-Forming Unit (CFU) Enumeration

After eight days of infection, the animals were euthanized and subjected to surface decontamination by brief immersion in 70% ethanol, followed by two washes in sterile PBS to prevent external contamination. Each fish was then fully macerated in 500 µL of PBS supplemented with 10% penicillin–streptomycin, yielding a uniform homogenate. Aliquots of 50 µL of the homogenate were plated onto Sabouraud dextrose agar plates. The plates were incubated at 37 °C for 24 to 72 h, after which colony counts were performed and fungal burden was calculated as colony-forming units per gram of tissue (CFU/g).

### Behavioral Analysis

Exploratory behavior was assessed eight days after infection using the novel tank test, following as described by Levin et al. [25], with some addptations. Levin et al. [25] used a plastic tank, and in this experiment was used a glass tank. Futhermore, in the Levin protocol was used the Noldus Image Analysis program, and in this study the behavioral parameters were recorded manually, using stopwatches and counters. Experiments were conducted in a controlled environment (25 ± 2 °C) between 9:00 a.m. and 1:00 p.m. to minimize circadian variability. The animals were placed in a trapezoid glass tank with de follow sizes: 27.9 cm at the top, 22.9 cm along the bottom, 15.2 cm high and 15.9 along the diagonal side, 6.4 cm wide at the top, and tapered to 5.1 cm at the bottom. The apparatus was filled whit 1.5-l of home tank water from the fish housing apparatus. After a 60-second habituation period, locomotor and exploratory activity was recorded for 5 min.

The following behavioral parameters were analyzed: latency to enter the top zone; time spent in the top, middle, and bottom zones of the tank; total freezing time; number of freezing episodes; number of entries into the top zone; and number of entries into the bottom zone [25].

### Assessment of Antioxidant Capacity by the ABTS Assay

The antioxidant activity of brain and liver homogenates was determined using the ABTS assay, which is based on the ability of samples to scavenge the stable free radical ABTS. For the assay, 190 µL of the ABTS solution were added to each well of a microplate, followed by the addition of 10 µL of the sample or standard (gallic acid). The mixture was gently homogenized and incubated for 30 min at 30 °C, protected from light, to allow development of the antioxidant reaction. After incubation, absorbance was measured at 734 nm using a microplate reader. Antioxidant activity was calculated by linear regression using a gallic acid standard curve. Results were expressed as nmol of gallic acid equivalents per mg of protein (ABTS, nmol GAE/mg protein).

### TNF-α Quantification

TNF-α levels were quantified in brain and liver homogenate supernatants from all experimental groups using a commercial ELISA kit (Zebrafish TNFα ELISA Kit, AFG Bioscience), according to the manufacturer’s instructions. The ELISA standard curve was performed in duplicate. Due to the limited amount of tissue obtained from individual zebrafish organs, samples were combined to generate three independent biological pools per experimental group prior to homogenization. Each pool was processed independently and analyzed in a single ELISA well for TNF-α determination. TNF-α concentrations were normalized to the total protein content previously determined in each homogenate and expressed as pg/mg protein.

### Histopathological Processing and Analysis

Samples were fixed in 10% buffered formalin and subsequently subjected to conventional histological processing, including dehydration, clearing, and paraffin embedding. After embedding, the fish were positioned laterally in cassettes, with the lateral surface in contact with the bottom of the cassette to ensure proper orientation during sectioning. Paraffin blocks were sectioned using a microtome at a thickness of 3 μm, obtaining transverse sections at three anatomical regions: (1) right eye (anterior region), (2) mouth, and (3) left eye (posterior region). The resulting slides were stained using the classical hematoxylin and eosin (H&E) method, following standardized protocols for dehydration, staining, and mounting. After staining, slides were examined under light microscopy for morphological tissue analysis.

In addition, histopathological analysis was performed based on the identification and semi-quantitative assessment of melanomacrophages and inflammatory processes in different tissues, including skin and gills. For this evaluation, an ordinal scoring system was used to compare the intensity of alterations among experimental groups. Alterations were classified according to their severity using the following criteria: 0 (absent), 1 (mild), 2 (moderate), and 3 (severe). This system allowed the attribution of a melanosis score (presence of melanomacrophages) and an inflammation score for each analyzed tissue.

Histopathological scores were assigned individually for each animal and tissue analyzed. Because the data were ordinal and non-parametric, results were expressed as median and interquartile range (IQR), allowing comparison of lesion severity distribution among experimental groups.

### Statistical Analysis

Statistical analyses were performed using GraphPad Prism 9 (GraphPad Software, San Diego, CA, USA). Data normality was assessed using the Shapiro–Wilk test. As several datasets did not meet the assumptions of normality and some experimental groups had relatively small sample sizes, non-parametric tests were applied. Comparisons among multiple groups were performed using the Kruskal–Wallis test followed by Dunn’s multiple comparisons test, when appropriate. Data are presented as median and interquartile range (IQR; P25–P75). A p value < 0.05 was considered statistically significant.

## Results

### Establishment of the Infection Model: Fungal Burden (CFU)

**Fig. 1:**
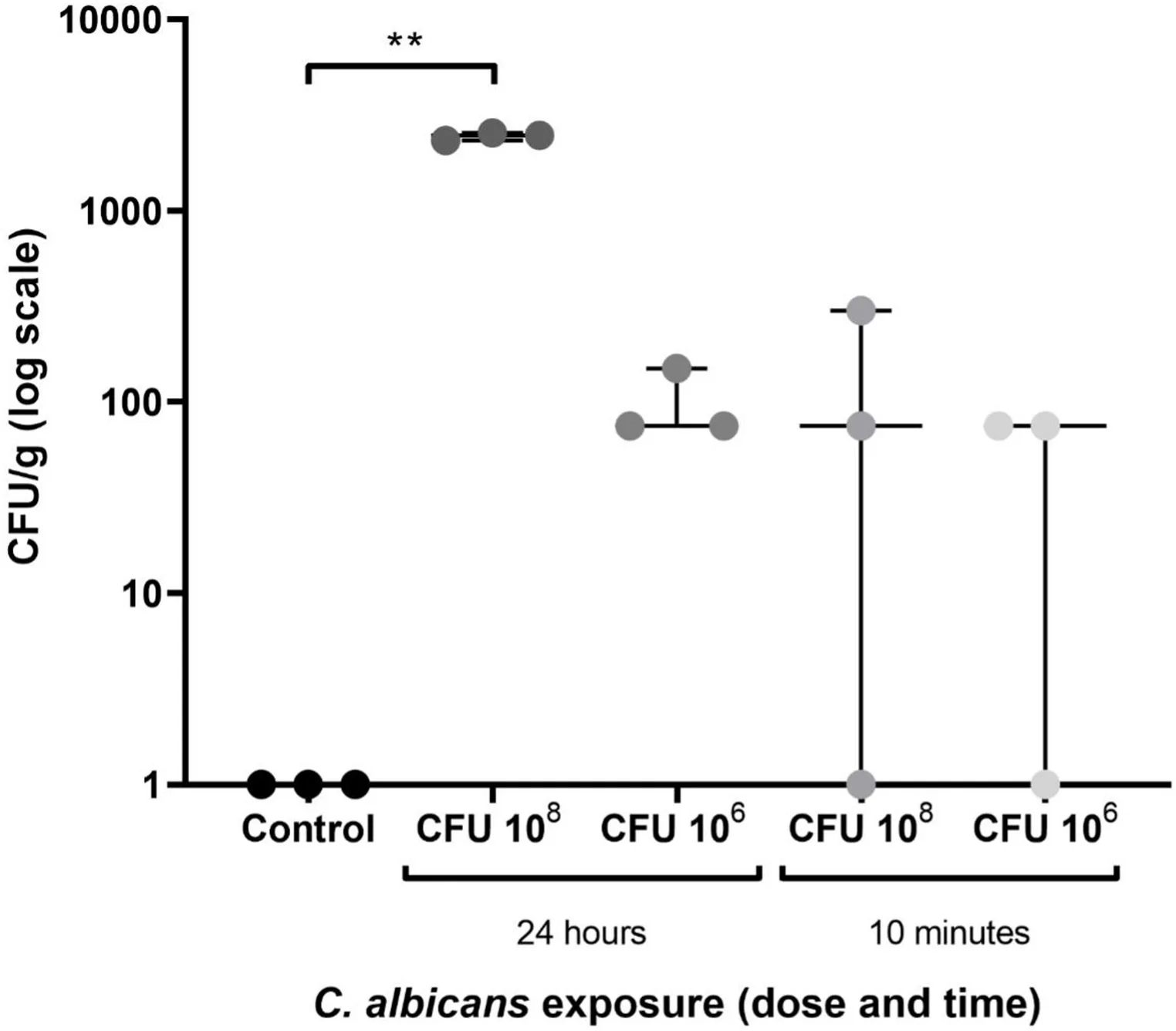
Fungal burden (CFU/g) in adult zebrafish (*Danio rerio*) following immersion exposure to different concentrations of *Candida albicans* and distinct exposure periods. Animals were exposed to *C. albicans* suspensions at concentrations of 1 × 10⁶ or 1 × 10⁸ CFU/mL for 10–24 h. Fungal burden was quantified from whole-animal homogenates 8 days post-exposure and expressed as CFU/g on a logarithmic scale. Individual animals are represented by dots, and data are presented as median and interquartile range (IQR) (*n* = 3 animals/group). Values equal to zero were plotted as 1 solely for logarithmic scale visualization. **p < 0.01

Animals were infected by immersion with different concentrations of *Candida albicans*. Exposure to a concentration of 1 × 10⁸ CFU/mL for 24 h resulted in a significant increase in fungal burden (CFU/g) (*p* < 0.0001) compared with the other tested conditions (1 × 10⁶ CFU/mL and different exposure times) (Fig. 1). No mortality was observed in any group during exposure or throughout the 8-day post-exposure period, supporting the sublethal nature of the proposed infection model.

### *C. albicans* Infection Alters Behavioral Parameters in Zebrafish

**Fig. 2:**
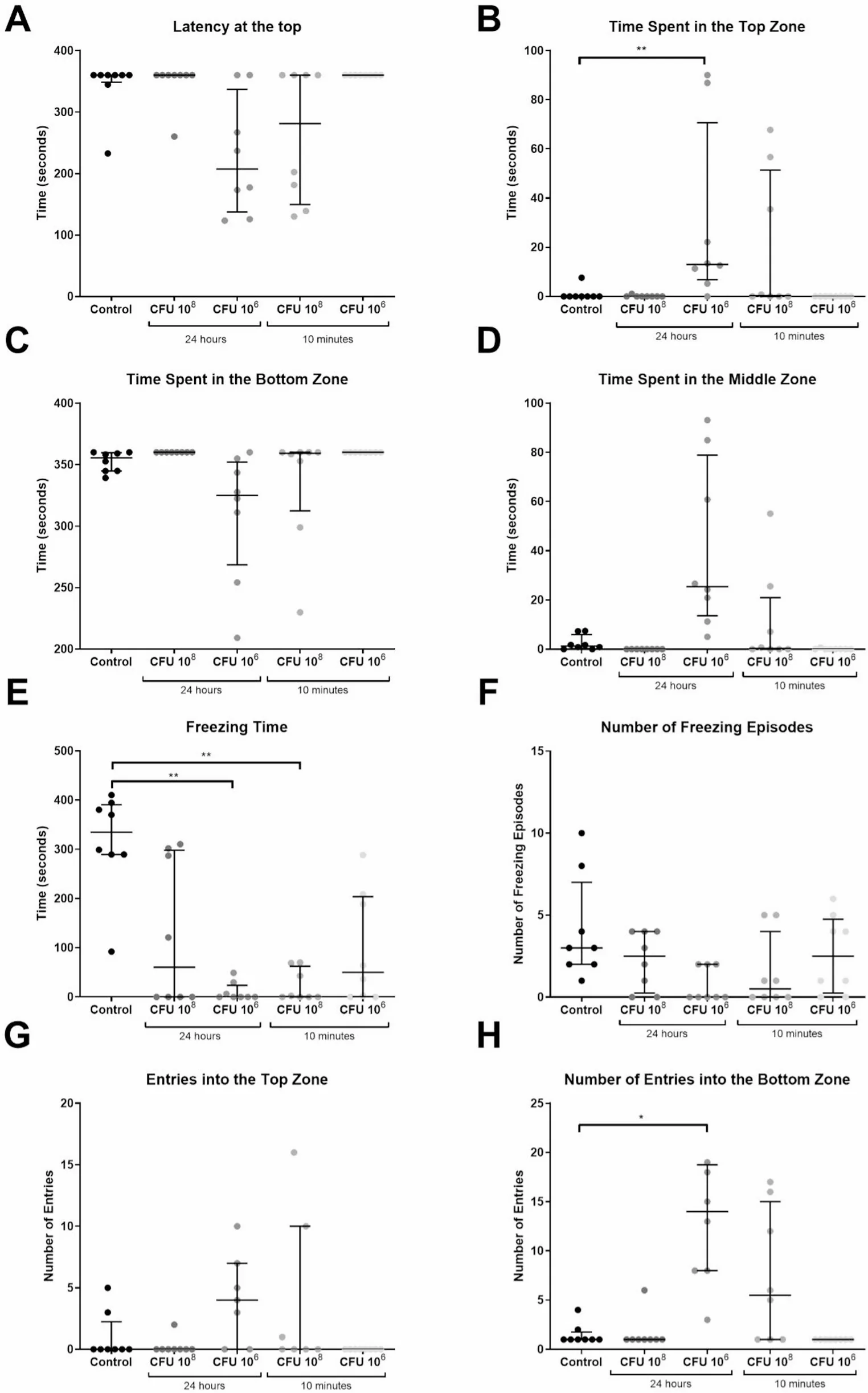
Behavioral assessment of adult zebrafish (*Danio rerio*) following immersion exposure to *Candida albicans*. Behavioral parameters were evaluated using the novel tank test and included: (**A**) latency to reach the top zone, (**B**) total time spent in the top zone, (**C**) total time spent in the bottom zone, (**D**) total time spent in the middle zone, (**E**) freezing time, (**F**) number of freezing episodes, (**G**) number of entries into the top zone, and (**H**) number of entries into the bottom zone. Individual animals are represented by dots, and data are presented as median and interquartile range (IQR) (*n* = 8 animals/group). *p < 0.05; **p < 0.01 indicates significant difference compared with the non-infected control group

Behavioral analysis in the novel tank test demonstrated selective alterations in exploratory and defensive-like behaviors following *Candida albicans* exposure (Fig. 2).

Animals exposed to 1 × 10⁶ CFU/mL for 24 h spent significantly more time in the top zone compared with non-infected controls (Fig. 2B). In parallel, this same group exhibited a significantly higher number of entries into the bottom zone (Fig. 2H).

Freezing behavior was also altered following fungal exposure. Animals exposed to 1 × 10⁸ CFU/mL for 24 h and those exposed to 1 × 10⁸ CFU/mL for 10 min showed significantly reduced freezing time compared with the control group (Fig. 2E).

Although not all behavioral parameters reached statistical significance, some infected animals exhibited greater variability in exploratory patterns and locomotor-related parameters compared with non-infected controls.

### Different Infection Concentrations Elicit Distinct Melanomacrophage and Inflammatory Responses in the Skin and Gills

**Fig. 3:**
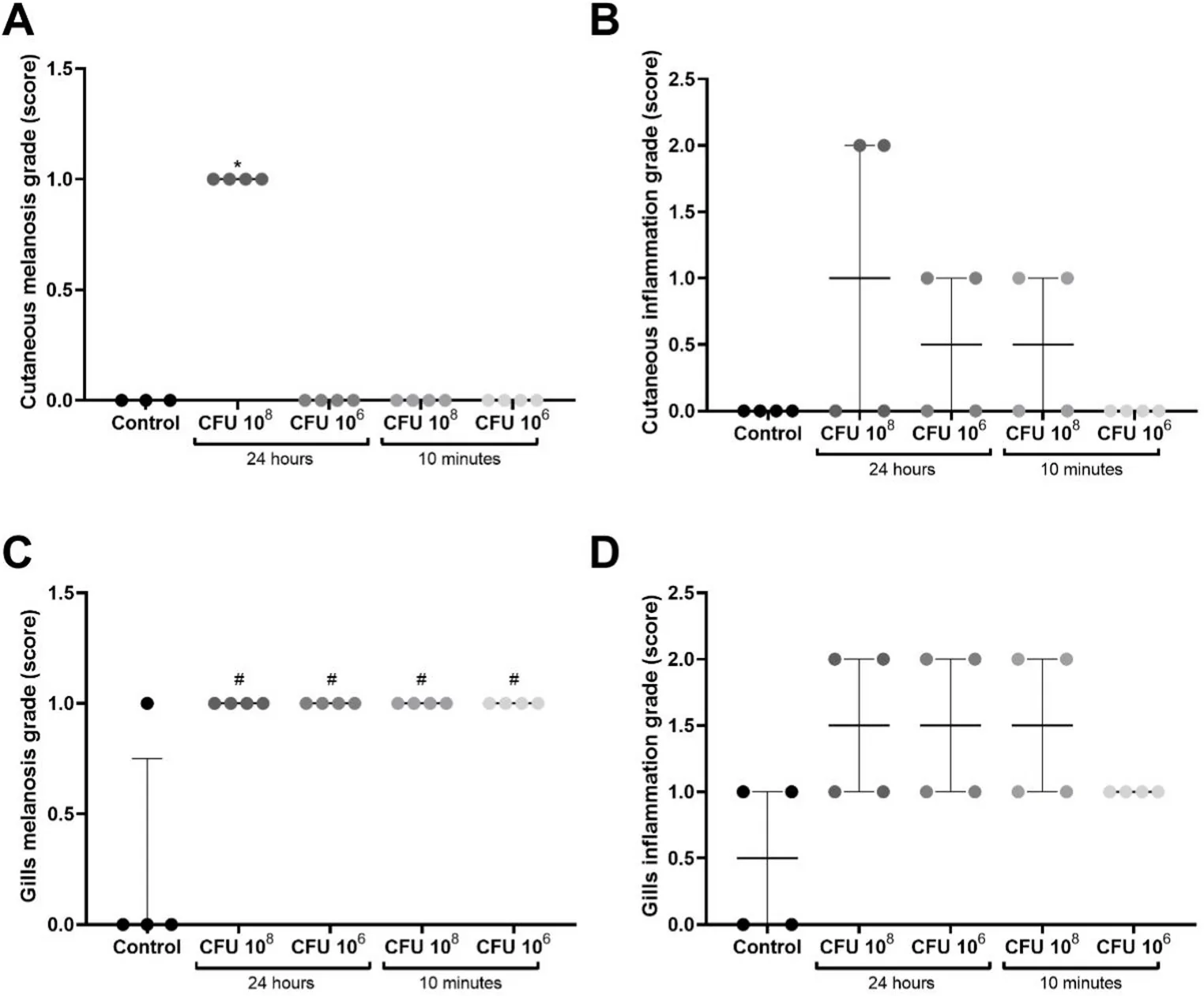
Histopathological evaluation of skin and gills from adult zebrafish (*Danio rerio*) following immersion exposure to *Candida albicans*. Semi-quantitative ordinal scores were assigned individually for each animal based on the intensity of melanomacrophage accumulation (melanosis grade) and inflammatory alterations observed in H&E-stained histological sections, using a scale ranging from 0 to 3. (**A**) Skin – melanosis grade, (**B**) Skin – inflammation grade, (**C**) Gills – melanosis grade, and (**D**) Gills – inflammation grade. Individual animals are represented by dots, and data are presented as median and interquartile range (IQR) (*n* = 4 animals/group). * indicates significant difference compared with all other groups (*p < 0.05). # indicates significant difference compared with the non-infected control group (#*p* < 0.05)

**Fig. 4:**
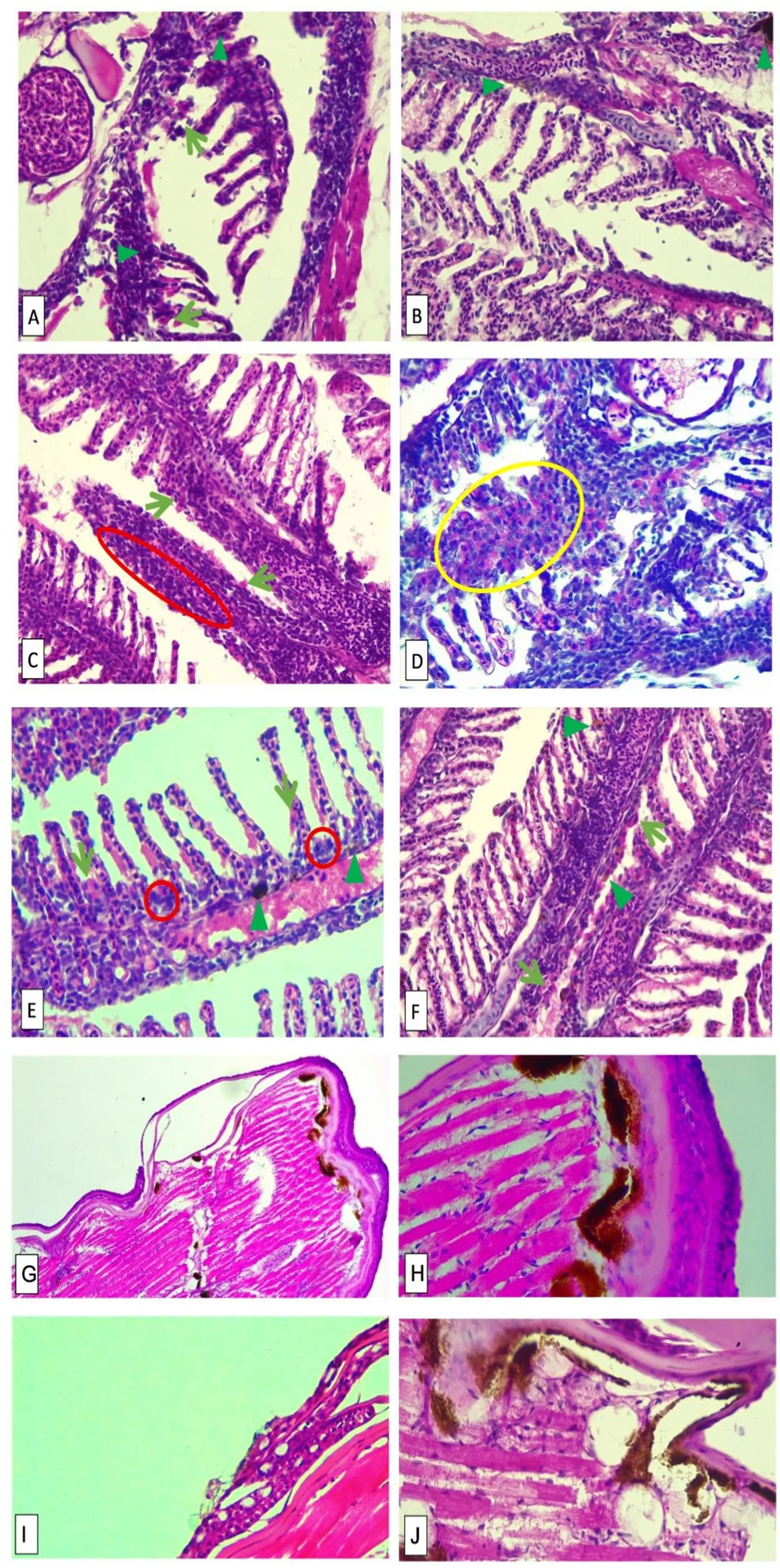
Representative histopathological findings in the gills (**A**-**F**) and skin (**G**-**J**) of adult zebrafish (*Danio rerio*) following immersion exposure to *Candida albicans* (H&E staining). (**A**) Mild inflammatory alterations characterized by the presence of melanomacrophages (arrowheads) and heterophils (arrows). (**B**) Mild melanomacrophage accumulation (arrowheads). (**C**) Moderate inflammatory infiltrate characterized by heterophils (arrows) and areas with lymphocytes (red circle). (D) Focal heterophilic infiltrate (yellow circle), indicating moderate inflammation. (**E**) Presence of melanomacrophages (arrowheads), heterophils (arrows), and focal lymphocytic infiltrates (red circles), consistent with moderate inflammation. (**F**) Moderate inflammatory alterations with melanomacrophage accumulation (arrowheads) and heterophilic infiltrates (arrows). (**G**) Melanomacrophage accumulation distributed within the dermis at the dermis–muscle interface. (**H**) Higher magnification of the melanomacrophage accumulation shown in panel A. (**I**) presence of Melanomacrophages covering the skin in the central area. (**J**) Melanomacrophages are observed in the dermis distributed at the interface with the muscle, close to adipocytes

Histopathological analysis demonstrated tissue-specific inflammatory alterations following Candida albicans exposure. Animals exposed to 1 × 10⁸ CFU/mL for 24 h exhibited a significantly higher degree of cutaneous melanosis compared with non-infected controls and others infected animals, although the alterations remained classified as mild (Fig. 3A). Histological examination revealed melanomacrophage accumulation within dermal regions and at the dermis–muscle interface (Fig. 4).

In the gills, all infected groups showed significantly higher melanosis scores compared with the control group, ranging from mild to moderate intensity (Fig. 3C). Histological evaluation demonstrated the presence of melanomacrophages, heterophils, and focal lymphocytic infiltrates, supporting local inflammatory modulation in branchial tissues (Fig. 4).

Although inflammatory scores in the skin and gills did not differ significantly among groups, some infected animals, particularly those exposed to 1 × 10⁸ CFU/mL for 24 h, presented higher inflammatory scores than non-infected controls, suggesting discrete and heterogeneous inflammatory alterations following fungal exposure (Fig. 3B, D).

### Infection Modulates Antioxidant Activity in the Brain and Liver

**Fig. 5:**
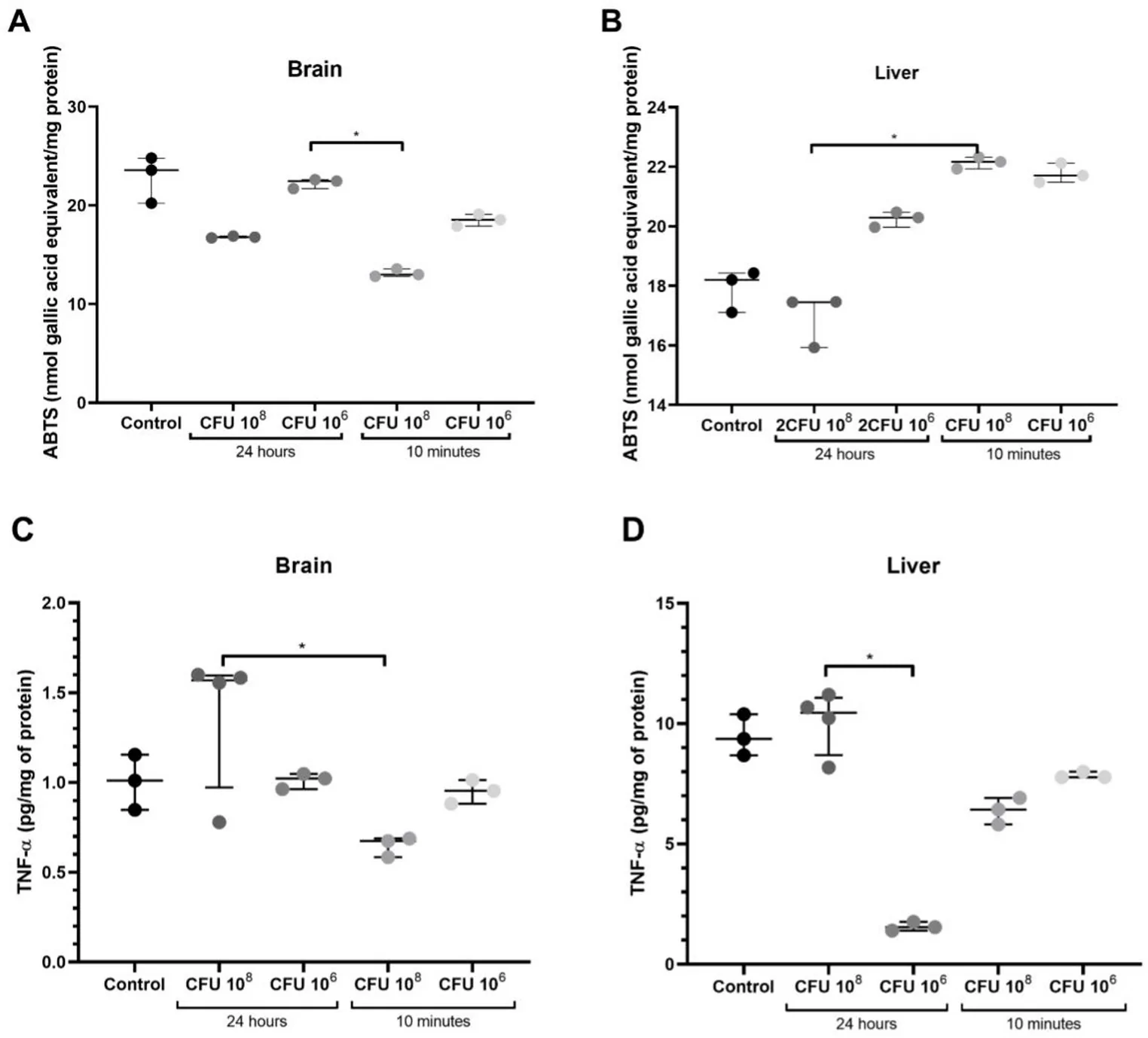
Otal antioxidant capacity assessed by the ABTS assay in brain (**A**) and liver (**B**), and TNF-α levels in brain (**C**) and liver (**D**) homogenates of adult zebrafish (*Danio rerio*) following immersion exposure to *Candida albicans*. Total antioxidant capacity is expressed as nmol of gallic acid equivalents per mg of protein, and TNF-α concentrations were quantified by ELISA and expressed as pg/mg of protein. Individual biological pools are represented by dots, and data are presented as median and interquartile range (IQR) (*n* = 3–4 biological pools/group). Each biological pool was generated by combining tissues from multiple animals within the same experimental group due to the limited amount of tissue obtained from individual organs. *p < 0.05 for comparisons among experimental groups

Assessment of total antioxidant capacity by the ABTS assay demonstrated tissue-specific modulation according to previous fungal exposure conditions. In the brain, animals previously exposed to *C. albicans* at 1 × 10⁶ CFU/mL for 24 h exhibited significantly higher antioxidant capacity on day 8 than those exposed to 1 × 10⁸ CFU/mL for 10 min (Fig. 5A). However, no significant differences were observed between infected groups and the non-infected control group.

In the liver, animals previously exposed to 1 × 10⁸ CFU/mL for 10 min showed significantly higher antioxidant capacity on day 8 than those exposed to the same fungal concentration for 24 h (Fig. 5B). Similarly, antioxidant capacity did not differ significantly between infected animals and the non-infected control group.

### Infection Reduces TNF-α Levels in the Liver and Brain

TNF-α levels were determined on day 8 after exposure to *Candida albicans*. In the liver, animals exposed to 1 × 10⁶ CFU/mL for 24 h showed significantly lower TNF-α levels compared with those exposed to 1 × 10⁸ CFU/mL for 24 h (Fig. 5D). In the brain, animals exposed to 1 × 10⁸ CFU/mL for 24 h exhibited higher TNF-α levels than those exposed to the same fungal concentration for 10 min (Fig. 5C). No significant differences were observed between infected groups and the non-infected control group in either tissue.

## Discussion

The zebrafish has emerged as a widely accepted model for studies of infectious diseases, including fungal infections, allowing visualization of host–pathogen interactions [12]. Although most studies rely on microinjection in larvae or adults, our results demonstrate that this species is also susceptible to environmental infection (immersion) by *Candida albicans*. We observed that exposure of adult zebrafish to a concentration of 1 × 10⁸ CFU/mL by immersion for 24 h was sufficient to establish a measurable infection, as evidenced by a significant increase in fungal burden, without inducing mortality. No deaths were recorded in any experimental group throughout the study. These findings characterize a sublethal infection model and indicate that this route represents an ecologically relevant and translationally valid platform for investigating the systemic effects of candidiasis.

Furthermore, fungal colonization can be influenced by factors such as the dynamic interplay between the infectious agent and the host immune response, phenotypic alterations in the yeast, including biofilm formation, mechanisms of antifungal drug resistance, yeast-to-hypha morphological transition, as well as infection sites with greater nutrient availability [26].

Several *Candida albicans* strains have been used in zebrafish larvae to investigate environmental conditions during infection, including markers of oxidative stress, alterations in arginine levels, and detection of morphological changes [12]. The literature predominantly focuses on microinjection protocols in embryos, larvae, and adults, often associated with reduced animal survival [27–29]. However, Wurster et al. described a protocol in which zebrafish larvae are infected with fungal pathogens after induction of epithelial cell loss, providing evidence that environmental exposure-based approaches can be used to investigate early fungal colonization and host–pathogen interactions [30].

Although *C. albicans* inocula were prepared after cultivation at 36 °C, exposure assays were conducted under zebrafish maintenance temperature (25 ± 2 °C). Temperature shifts may influence fungal physiology and virulence-associated traits. However, the significant increase in fungal burden observed in animals exposed to the highest inoculum for 24 h indicates that fungal cells remained viable and capable of proliferation under the experimental conditions. Moreover, biological alterations observed in other exposure groups suggest that host–pathogen interactions occurred even under shorter exposure periods or lower inoculum. Nevertheless, future studies comparing fungal preconditioning at zebrafish-relevant temperatures may further refine the interpretation of host–fungal interactions in this experimental model [13].

The behavioral alterations observed in the novel tank test suggest that exposure to Candida albicans modulated exploratory and defensive-like behaviors in adult zebrafish. In animals exposed to 1 × 10⁶ CFU/mL for 24 h, reduced latency to reach the top zone and altered exploration patterns suggest modulation of exploratory behavior following fungal exposure. In addition, the reduction in freezing behavior observed in infected groups may reflect neurobehavioral modulation associated with neuroimmune signaling, locomotor alterations, or delayed inflammatory effects rather than a direct anxiolytic-like response. Interestingly, these behavioral changes did not follow a classical dose–response pattern, reinforcing the complexity of the relationship between peripheral fungal exposure and central behavioral outcomes. Since behavioral assessments were performed 8 days after exposure, the observed effects likely represent delayed neurobehavioral consequences rather than immediate responses to the immersion period itself.

Previous studies in zebrafish have demonstrated associations between exploratory and social behaviors and inflammatory cytokine profiles, supporting the concept that neuroimmune interactions may influence behavioral responses following immune or pathogenic stimuli. For example, altered exploratory behavior has been associated with increased IL-1β expression and reduced IL-10 levels, reinforcing the relationship between inflammatory signaling and behavioral modulation [31].

There are few studies regarding the behavioral influence of *C. albicans* infection in zebrafish. In contrast, in murine models, *C. albicans* colonization has been associated with dysregulation of hypothalamic-pituitary-adrenal (HPA) axis-related neuroendocrine responses, including increased corticosterone production, potentially linked to gut microbiota dysbiosis [32]. Although these findings were obtained in mammals under different exposure conditions, they provide complementary mechanistic evidence supporting possible interactions between fungal exposure, neuroendocrine signaling, and behavioral modulation. Nevertheless, zebrafish possess a conserved hypothalamic–pituitary–interrenal (HPI) axis functionally analogous to the mammalian HPA axis.

Thus, fungal infection may contribute to stress-related neurobehavioral alterations through interactions involving the gut microbiota, neuroendocrine pathways, and immune signaling. Additionally, it has been suggested that modulation of the gut microbiota with probiotics such as *Bifidobacterium longum* and *Lactobacillus paracasei* can reduce corticosterone levels and influence central neurotransmitters such as serotonin and dopamine, thereby reversing the anxiety-like behavior induced by *C. albicans* [33, 34].

Another relevant finding in murine models is the negative regulation of the HPA axis via endocannabinoid receptors, as the neuroendocrine consequences of *C. albicans* infection include modulation of these receptors. Colonization by *C. albicans* reduces the activation of anandamide and CB1 receptors, resulting in hyperactivation of the HPA axis and thereby promoting stress and anxiety [32, 35].

In addition, the immune system plays a key role in regulating neuronal plasticity, learning, and behavior, as low concentrations of cytokines such as TNF-α, IL-1, and IL-6 are essential for the maintenance of synaptic plasticity, neurogenesis, and proper functioning of neural circuits. However, during infectious processes, this balance may be disrupted, leading to alterations in cytokine levels and, consequently, to neurophysiological and behavioral dysfunction [36].

In the present study, TNF-α levels were not uniformly reduced or increased, but instead varied according to tissue compartment, fungal burden, and exposure duration, suggesting a context-dependent host inflammatory response. Tumor necrosis factor-alpha (TNF-α) is a central mediator of innate antifungal immunity and is rapidly produced after recognition of Candida albicans by monocytes, macrophages, and neutrophils through pattern-recognition receptors, including Toll-like receptors and C-type lectin receptors such as Dectin-1. In addition, TNF-α contributes to early inflammatory activation, leukocyte recruitment, and fungal killing during candidiasis [37].

Although *Candida albicans* is known to activate inflammasome pathways, particularly NLRP3, during the early innate immune response [38, 39], inflammasome signaling and TNF-α production are regulated through partially distinct mechanisms and may display different temporal kinetics. Therefore, acute inflammasome activation does not necessarily imply sustained TNF-α elevation at later stages of infection [37, 40, 41]. Because TNF-α was measured on day 8 post-exposure in the present study, the observed profile likely reflects a delayed inflammatory state rather than the initial innate response to fungal recognition.

In the liver, TNF-α levels were lower after exposure to 1 × 10⁶ CFU/mL for 24 h than after 1 × 10⁸ CFU/mL for 24 h, suggesting that prolonged higher fungal burden may sustain stronger inflammatory signaling. This interpretation is consistent with the recognized role of the liver as a central immunometabolic organ involved in pathogen sensing, innate immune surveillance, and systemic inflammatory regulation [42].

In the brain, animals exposed to 1 × 10⁸ CFU/mL for 24 h exhibited higher TNF-α levels than those exposed to the same inoculum for 10 min. Because TNF-α was measured on day 8 after exposure, this finding likely reflects a delayed inflammatory outcome rather than an acute cytokine response. Although the underlying mechanisms were not directly investigated, this interpretation is consistent with reports showing that *Candida* species can reach the central nervous system and induce local immune activation [43].

However, because all analyses were performed on day 8 post-exposure, these findings likely represent late immunological outcomes rather than acute cytokine kinetics immediately after infection.

In addition to inflammatory signaling, systemic fungal exposure may also contribute to cerebral oxidative stress through excessive production of reactive oxygen species (ROS), a process that may compromise microglial function [44] and alter neuroimmune dynamics even in the absence of direct central nervous system invasion. Under redox imbalance conditions, microglia may shift toward hypoactive or dysfunctional states, resulting in reduced cytokine release [44].

Within the inflammatory process triggered by fungal infection, melanomacrophage centers present in zebrafish can be analyzed. These structures are considered analogous to secondary lymphoid organs in mammals and play a role in inflammatory processes such as antigen recognition and regulation of the infectious response. Melanin pigments within melanomacrophages, in addition to absorbing and dispersing light, are capable of neutralizing free radicals and directly influencing the immune system, thereby contributing to the prevention of excessive inflammatory damage [45]. Melanomacrophages express major histocompatibility complex (MHC) class II molecules, which activate T lymphocytes. In addition, they express Toll-like receptors and C-type lectin receptors, which induce the expression of pro-inflammatory cytokines such as TNF-α, IL-1β, and IL-6 [46].

Animals exposed to 1 × 10⁸ CFU/mL for 24 h exhibited a higher degree of cutaneous melanomacrophage accumulation, accompanied by mild inflammatory alterations in the skin. In addition, all infected groups showed mild to moderate melanomacrophage presence in the gills, together with discrete inflammatory infiltrates characterized by heterophils and focal lymphocytic accumulation. Although inflammatory scores did not differ significantly among groups, infected animals frequently exhibited higher inflammatory scores than non-infected controls, particularly following exposure to 1 × 10⁸ CFU/mL for 24 h.

Since melanomacrophage phagocytic activity is enhanced during infectious and inflammatory processes, the increased presence of these cells in the skin and gills of zebrafish may reflect local innate immune activation following fungal exposure [13, 47].

Products of the melanin synthesis pathway, such as the pituitary-derived α-melanocyte-stimulating hormone (α-MSH), have also been associated with immunomodulatory effects, including suppression of pro-inflammatory cytokine production and inhibition of NF-κB signaling pathways [48, 49]. Thus, melanomacrophage-associated responses may contribute not only to local inflammatory containment but also to modulation of inflammatory signaling during host–fungus interaction.

Although TNF-α plays important physiological roles in the central nervous system, including regulation of synaptic plasticity and memory processes, its dysregulation may also contribute to neurotoxic effects, cognitive impairment, and behavioral alterations [36]. In addition, TNF-α is known to modulate AMPA receptor trafficking and influences glutamatergic neurotransmission; therefore, changes in TNF-α levels may affect neural circuits involved in risk assessment, defensive behavior, and responses to environmental stimuli [50, 51]. In the present study, the higher brain TNF-α levels observed after prolonged exposure to the highest fungal inoculum may indicate that sustained host–fungal interaction influences late neuroimmune signaling. Furthermore, systemic infections can induce morphofunctional changes in microglia, altering cytokine secretion profiles and contributing to the modulation of central neural circuits [36].

Oxidative and antioxidant biomarkers have been widely employed in zebrafish models to evaluate tissue-specific redox modulation under physiological and pathological conditions, particularly in the liver and brain, which exhibit distinct sensitivities and adaptive responses to oxidative and inflammatory stimuli [52]. The liver is a key organ in the response to oxidative stress and is capable of responding to danger signals through the activation of endogenous antioxidant systems [53]. Thus, mild inflammatory stimuli may induce compensatory alterations in hepatic antioxidant capacity. Since ABTS measurements were performed on day 8 post-exposure, the observed findings likely represent delayed tissue-specific antioxidant modulation associated with previous fungal exposure conditions rather than immediate oxidative responses during the immersion period itself.

In our study, lower concentrations of *C. albicans* or shorter exposure times were associated with higher antioxidant activity (ABTS) in the liver. The ABTS assay has been successfully applied in tissue homogenates to assess changes in total antioxidant capacity under inflammatory conditions, reinforcing its relevance for investigating tissue-specific redox modulation following pathogenic stimuli [54]. Importantly, although significant differences were observed among infected groups, ABTS values did not differ significantly from the non-infected control group, suggesting relative modulation of antioxidant capacity rather than overt oxidative dysfunction. Regarding hepatic inflammation, TNF-α modulation depended on fungal burden, with lower values in animals exposed to 1 × 10⁶ CFU/mL for 24 h compared with 1 × 10⁸ CFU/mL for the same period. These findings suggest that a low-intensity fungal exposure may be associated with tissue-specific adaptive hepatic modulation, potentially involving relative changes in antioxidant capacity and TNF-α signaling mediated by fungal PAMP recognition through pattern recognition receptors [37].

*Candida albicans* is capable of negatively modulating the host’s inflammatory response by suppressing pro-inflammatory cytokines such as IL-12p70 and inducing anti-inflammatory profiles, including IL-35. These effects promote the polarization of macrophages toward M2 phenotypes, which produce fewer inflammatory cytokines and foster immune tolerance. Thus, the fungus may attenuate Th1 responses and maintain a symbiotic relationship, preventing excessive inflammatory activation [55].

Reductions in TNF-α levels have also been reported in other host–*Candida* interaction models. For instance, Wang et al. [56] demonstrated that soluble factors such as mannose-binding lectin (MBL) can suppress TNF-α production in a dose-dependent manner in macrophages stimulated with *C. albicans*. These findings reinforce the notion that the inflammatory response to the fungus can be negatively modulated depending on the immunological context, which is consistent with the decrease in TNF-α observed in our model, particularly under milder or prolonged infection conditions.

To the best of our knowledge, there are no specific reports describing a reduction in hepatic TNF-α levels in models of mild or prolonged *C. albicans* infection, particularly in zebrafish. Thus, our data suggest that low or moderate fungal exposure may be associated with relative attenuation of TNF-α signaling in the zebrafish liver.

Our study demonstrates, for the first time, that adult zebrafish can be reproducibly, sublethally, and biologically relevantly infected with *Candida albicans* via immersion, enabling the simultaneous investigation of behavioral, immunological, oxidative, and histopathological alterations. We show that environmental exposure to *C. albicans* was associated with tissue-specific modulation of behavioral, inflammatory, and antioxidant parameters, which varied according to fungal burden and exposure duration. Importantly, these alterations and host–pathogen interactions are complex and vary substantially according to infection parameters, such as inoculum size and duration of exposure.

Thus, this work establishes a new experimental paradigm for the study of candidiasis in teleosts and opens avenues for deeper mechanistic investigations, the identification of neuroimmune biomarkers, and the screening of therapeutic candidates. As limitations, we acknowledge the lack of detailed molecular characterization of the pathways involved, as well as the need to explore longer exposure periods, additional fungal concentrations, and the virulence of different *C. albicans* strains. In addition, behavioral analyses were performed using a relatively limited number of animals per group, which may have reduced the power to detect subtle behavioral differences among experimental conditions. Additionally, TNF-α quantification was performed using pooled brain and liver samples due to the limited amount of tissue obtained from individual zebrafish organs. Although the ELISA standard curve was performed in duplicate according to the manufacturer’s instructions, each biological pool was analyzed in a single well, preventing the assessment of technical assay variability within individual samples.

Future studies employing larger sample sizes and technical replicates may further strengthen the robustness of these findings. Furthermore, these studies may also investigate inflammasome-associated responses, including IL-1β production and gasdermin-mediated pyroptotic pathways, together with real-time monitoring of fungal progression and deeper characterization of immunoneurological mechanisms involved in *C. albicans* infection, and the testing of antifungal agents or immunomodulatory compounds. The model proposed herein positions adult zebrafish as a promising translational platform for investigating candidiasis and fungus–host interactions under environmentally relevant conditions.

## Data Availability

No datasets were generated or analysed during the current study.
